# CCAAT/Enhancer Binding Protein β (C/EBPβ) Isoforms as Transcriptional Regulators of the Pro-Invasive *CDH3*/P-Cadherin Gene in Human Breast Cancer Cells

**DOI:** 10.1371/journal.pone.0055749

**Published:** 2013-02-06

**Authors:** André Albergaria, Carlos Resende, Ana Rita Nobre, Ana Sofia Ribeiro, Bárbara Sousa, José Carlos Machado, Raquel Seruca, Joana Paredes, Fernando Schmitt

**Affiliations:** 1 Cancer Genetics Group, Institute of Molecular Pathology and Immunology of Porto University (IPATIMUP), Porto, Portugal; 2 Department of Pathology, Medical Faculty of Porto University, Porto, Portugal; 3 Institute of Biomedical Sciences of Abel Salazar (ICBAS), Porto, Portugal; Ghent University, Belgium

## Abstract

P-cadherin is a cell-cell adhesion molecule codified by the *CDH3* gene, which expression is highly associated with undifferentiated cells in normal adult epithelial tissues, as well as with poorly differentiated carcinomas. In breast cancer, P-cadherin is frequently overexpressed in high-grade tumours and is a well-established indicator of aggressive tumour behaviour and poor patient prognosis. However, till now, the mechanisms controlling *CDH3* gene activation have been poorly explored. Since we recently described the existence of several CCAAT/Enhancer Binding Protein β (C/EBPβ) transcription factor binding sites at the *CDH3* promoter, the aim of this study was to assess if the distinct C/EBPβ isoforms were directly involved in the transcriptional activation of the *CDH3* gene in breast cancer cells. DNA-protein interactions, mutation analysis and luciferase reporter assay studies have been performed. We demonstrated that C/EBPβ is co-expressed with P-cadherin in breast cancer cells and all the three isoforms function as transcriptional regulators of the *CDH3* gene, directly interacting with specific regions of its promoter. Interestingly, this transcriptional activation was only reflected at the P-cadherin protein level concerning the LIP isoform. Taken together, our data show that *CDH3* is a newly defined transcriptional target gene of C/EBPβ isoforms in breast cancer, and we also identified the binding sites that are relevant for this activation.

## Introduction

The molecular changes that occur during breast cancer progression, which include the amplification/overexpression of transcription factors, can disrupt the delicate balance between cell proliferation, differentiation and apoptosis. C/EBPβ is one of those transcription factors, which has been implicated in cell cycle regulation, playing an important role in mammary gland development and oncogene-induced breast tumorigenesis [1]–[4]. Encoded by an intronless gene, C/EBPβ is expressed as distinct protein isoforms, which can accomplish distinct biological and regulatory functions, ultimately leading to gene transactivation [5]. The longer C/EBPβ proteins (liver-enriched transcriptional activating proteins, LAP1 and LAP2) regulate proliferation and differentiation of many cell types [6]; the shorter protein product (liver-enriched transcriptional inhibitory protein, LIP) lacks the transactivation domain and acts mainly as a dominant-negative [7]. AS LAP isoforms, LIP also binds to the consensus sequences within genomic DNA, sometimes even with a higher affinity than the other C/EBPβ isoforms [6], [7]. In fact, LIP inhibits the transcriptional activity of LAPs by competing for the same consensus binding sites or by forming inactive heterodimers with them. However, some emerging evidence suggest that LIP can also act as a transcriptional activator in some cellular contexts [5].

In breast, C/EBPβ most likely contributes to tumorigenesis through significant elevations in the LIP∶LAP ratio, mostly observed in ER-negative, highly proliferative and metastatic mammary tumours, usually associated with a poor patient prognosis [8]. Indeed, LIP isoform overexpression has been associated to a lack of contact inhibition, resulting in proliferation and *foci* formation in epithelial breast cancer cell lines [9]. It has been hypothesized that aberrant expression of C/EBPβ-LIP isoform may contribute to an increased growth rate and result in a more proliferative and aggressive breast carcinoma.

P-cadherin, a classical cadherin encoded by the *CDH3* gene [10], has been explored by our group for several years and has been also extensively associated with breast tumour aggressiveness. This protein was found to be aberrantly expressed in 20–40% of invasive ductal carcinomas, being strongly associated with proliferative lesions of high histological grade, decreased cell polarity and poor patient survival [11]–[16]. At the *in vitro* level, we demonstrated that P-cadherin overexpression induces invasion [14], motility and migration of wild-type E-cadherin expressing breast cancer cells, through the secretion of pro-invasive factors, such as matrix metalloproteinase (MMP)-1 and MMP-2 [17]. In fact, P-cadherin-associated functions in breast cancer have been widely studied, which reflects the growing importance of this cadherin in human breast cancer biology and prognosis.

However, the mechanisms controlling its overexpression in breast cancer have only recently started to be unrevealed [11], [18]. In non-cancer models, *CDH3* promoter was shown to be genetically regulated through direct binding of transcription factors, such as p63 [19] and β-catenin [20]. Gorski and collaborators also demonstrated that BRCA1 and c-Myc form a repressor complex on *CDH3* promoter and on other promoters of specific basal genes, representing a potential mechanism to explain the overexpression of key basal markers in BRCA1-deficient breast tumours [21]. Additionally, we established a direct link between P-cadherin overexpression and the lack of oestrogen receptor (ER)-signalling in breast cancer cells, categorizing *CDH3* as a putative ER-repressed gene [14]. In 2010, we described a regulatory mechanism whereby a selective ER-downregulator is able to up-regulate P-cadherin expression in MCF-7/AZ breast cancer cells through chromatin remodelling at *CDH3* promoter level [18]. This epigenetic process was accomplished by the induction of high levels of the active chromatin mark H3K4me2 and a consequent de-repression of the *CDH3* promoter, which exposed a high number of putative C/EBPβ transcription binding sites [18]. The induction of *CDH3* promoter activity by C/EBPβ was also confirmed by reporter assays, as well as its expression association with worse prognosis of breast cancer patients [18].

However, since the mechanistic link and the consequent transcriptional regulatory relevance of C/EBPβ proteins on *CDH3* gene were not demonstrated, in the present study we revealed that C/EBPβ isoforms are indeed transcriptional regulators of P-cadherin, directly interacting with conserved and specific regions of the *CDH3* promoter. Interestingly, we show that this transcriptional activation is reflected in the P-cadherin protein levels, especially for the LIP isoform. We conclude that *CDH3* is a newly defined transcriptional target gene of C/EBPβ in breast cancer.

## Materials and Methods

### Antibodies

The following primary anti-human antibodies were used for Western Blot and/or Immunohistochemistry against: P-cadherin (BD Transduction Biosciences, Lexington, KY), C/EBPβ (Santa Cruz Biotechnology, CA), β-actin (Santa Cruz Biotechnology) and β-tubulin (Sigma-Aldrich, St. Louis, NO). Technical conditions are described in Table S1 (Supporting Information). Anti-mouse and anti-goat horseradish peroxidase-conjugated secondary antibodies were used for WB [HRP-conjugated, dilutions: 1∶2000] (Santa Cruz Biotechnology). For chromatin immnunoprecipitation (ChIP) assays, the following antibodies were used: anti-C/EBPβ (C-19, Santa Cruz Biotechnology), and two control IgGs (Active Motif, CA and Santa Cruz Biotechnology).

### Promoter Vectors and cDNA Constructs

The pLENTI-C/EBPβ expression vectors (C/EBPβ-LAP1, C/EBPβ-LAP2 and C/EBPβ-LIP) were generated according to the human *CEBPB* nucleotide sequence obtained from Ensembl and Pubmed databases. Oligonucleotide primer sequences for LAP1, LAP2, and LIP isoforms are listed in Table S2 (see Supporting Information).

*CEBPB* cDNA was obtained from total RNA extracted from the gastric cancer cell line AGS, and amplified for each *CEBPB* isoform using HotStart Taq DNA Polymerase (Qiagen, Cambridge, MA). Amplification was performed for 35 cycles as follows: denaturation at 95°C for 1 minute, annealing at 60°C for LAP1 and LAP2 and 58°C for LIP for 1 minute, and extension at 68°C for 2 minutes per cycle. PCR products for each isoform were separated by electrophoresis in a 1.5% agarose gel and bands were sequenced using the ABI Prism Dye Terminator Cycle Sequencing Kit (Perkin-Elmer, Beaconsfield, UK). To validate the isoforms nucleotide sequence, amplified products were purified through Sepharose (GE Healthcare, Waukesha, WI) and sequenced on both strands on an ABI Prism 3100 automated sequencer (Perkin-Elmer). PCR products were inserted into the mammalian expression vector pLENTI6/V5 Directional (Invitrogen, Ltd, Paisley, UK), using manufacturer instructions, and incorporated into chemically competent TOP10 *E. coli* (Invitrogen). Transformed bacteria were grown overnight in ampicillin-supplemented LB-Agar (Applichem, Germany). Plasmid DNA from transformed *E. coli* cells was sequenced to check the orientation and nucleotide sequence for each *CEBPB* isoform.

The human full-length *CDH3*-luciferase vector was generated by our group, as previously described [18]. Normalization pRL-CMV Renilla Luciferase Control Reporter Vector was purchased to Promega (Promega Corporation, Madison, WI).

### Immunohistochemistry

Double immunostaining for C/EBPβ and P-cadherin was performed in 3 µm sections of 23 formalin-fixed paraffin-embedded (FFPE) invasive breast carcinomas that have previously showed strong expression of both proteins, in order to illustrate their consistent cellular co-localization. Standard immunohistochemistry was performed as previously described [16]. For the reaction, we used the Envision G2 Double-stain (DakoCytomation, Glostrup, Denmark), according to manufacturer instructions. Specific conditions used for C/EBPβ and P-cadherin are listed in Table S1. FFPE sections from normal breast gland, skin or normal gastric mucosa were used as positive controls for C/EBPβ and P-cadherin. Negative controls were performed by replacing the primary antibody with PBS/non-immune serum.

The present study was conducted under the national regulative law for the usage of biological specimens from tumour banks, where the samples are exclusively available for research purposes in the case of retrospective studies (National Regulative Law number 12/2005 – I Serie-A, n°. 18–26^th^ January, 2005).

### Cell Culture

Human breast cancer cell line MCF-7/AZ was kindly provided by Prof. Marc Mareel (Ghent University, Belgium) [22], while BT-20 cells were purchased to American Type Culture Collection (ATCC, Manassas, VA). Cell lines were routinely maintained at 37°C, 5% CO_2_, in the following media (Invitrogen): 50% DMEM/50% HamF12 (MCF-7/AZ), or only DMEM (BT-20). All media contained 10% of heat-inactivated foetal bovine serum (Greiner Bio-one, Wemmel, Belgium), 100 IU/mL penicillin and 100 mg/mL streptomycin (Invitrogen).

### Transient Transfection

For gene reporter assays, cells were grown in 96-well plates to 60–70% confluence and transfection was done using the liposome-mediated FuGENE 6 transfection reagent (Roche Diagnostic GmbH, Mannheim, Germany), prepared according to the manufacturer's instructions. A ratio of FuGENE/DNA of 3∶1 was used. For protein expression assays, cells were grown in 6-well plates to 60% confluence. Transient transfections of C/EBPβ expression vectors were done using Lipofectamine 2000 (Invitrogen), with a ratio of Lipofectamine/DNA of 3∶1 and prepared according to the manufacturer's instructions.

For knock-down assays, cells were transiently transfected at 60% confluence with specific siRNA for C/EBPβ (100 nM, FlexiTube siRNA – Hs_C/EBPβ 5-Qiagen) using Lipofectamine 2000 (Invitrogen), according to the manufacturer's procedure. Maximum C/EBPβ knock-down was achieved after 48 h of incubation. A siRNA with no homology to any gene was also used as a negative control.

### *CDH3*-luciferase Reporter Gene Analysis

Cells were co-transfected with pGL3-*CDH3*/luc promoter vector (20 ng) and with pRL-CMV Renilla vector (5 ng). For promoter analysis, 24 hours after transfection, cells were washed twice in PBS-cold and lysed for firefly/Renilla luciferase assays, using the Luclite Reporter Gene Assay System (Perkin Elmer), according to the manufacturer. Luciferase bioluminescence from Renilla was measured using native coelenterazine substrate reagent (Lux Biotechnology, Edinburgh, UK). Individual transfection experiments were repeated at least three times and in quadruplicate per transfection condition. Empty pGL3-basic vector and pGL3/luc-Control (pLUC) vector (Promega) were included as controls in all *CDH3*-reporter assays. Luminescence was read using the Wallac/Perkin Elmer-1450-028 Trilux Microbeta (Perkin Elmer) plate reader, and the results are shown as a mean of relative light units (RLU), which was calculated by the ratio between the luminescence signal emitted from luciferase and the luminescence signal obtained by the Renilla (normalization).

### Western Blot

Cells were lysed and the concentration of total protein was determined by Bradford quantification. Western Blot was performed as earlier described [17], [18]. For MCF-7/AZ cell line, due to its lower expression of P-cadherin, 50 µg of total protein lysate has been loaded; for BT-20, due to its P-cadherin overexpression, the gel loading was done only with 20 µg of protein lysate. Membranes were incubated with primary antibodies according to the conditions described in Table S1.

### Site-Directed Mutagenesis

All the C/EBPβ binding sites mutations in *CDH3* promoter were performed in order to impair the binding of any predicted transcription factor: bioinformatic prediction tools were used to blast all point mutated sequences. To introduce point mutations in the *CDH3* promoter region, the QuickChange Site-directed Mutagenesis Protocol (Stratagene, Cedar Creek, USA) was followed, and the oligos used are listed in Table S2. The PCR cycles were set as follows: 95°C for 30 seconds; 16 cycles of 95°C for 30 seconds, 55°C for 1 minute, and 68°C for 5 minutes. Following PCR reaction, products were incubated with DpnI (1 hour at 37°C) and transformed into *E-coli* competent cells (Stratagene). All mutated plasmids were checked by sequencing and primer sequences are also listed in Table S2.

### Chromatin Immunoprecipitation (ChIP) Assay

For chromatin immunoprecipitation of the endogenous *CDH3* promoter regions in MCF-7/AZ cells, the ChIP-IT™ kit (Active Motif) was used and the assay was performed according with the manufacturer's procedures. Briefly, cells (4.5×10^7^) were fixed with 1% formaldehyde in culture medium for 10 minutes. Fixation was stopped by incubating the cells for 5 minutes with a 1× Glycine Stop-Fix Solution, homogenized and centrifuged. The cell-pellets were resuspended in a shearing buffer and sonicated into chromatin fragments of 200–1500 bp in length. To reduce non-specific background, sonication-sheared lysates were pre-cleared with Protein G beads. The sheared chromatin lysates were incubated with 5 µg of C/EBPβ antibody or with a control rabbit IgG, overnight at 4°C, and immunoprecipitated with Protein G beads (2 hours at 4°C). The precipitated DNA-protein complex was washed 7 times, eluted, incubated for 8 hours at 65°C in a reverse cross-link buffer, and digested with proteinase K for 2 hours at 42°C. The resultant DNA was purified, resuspended in DEPC H_2_O and quantified by real-time qPCR amplification. The PCR primers sequences used in this amplification are listed in Table S2.

For chromatin Immunoprecipitation in BT-20 cells and in an invasive breast carcinoma highly positive for P-cadherin and C/EBPβ, the Magna ChIP G Kit (Millipore) was used, according to manufacturer's protocol. Basically, the essential steps applied for BT-20 cells were the same as the ones used for MCF-7/AZ cells, differing only in the use of protein G magnetic beads instead of non-magnetic beads for simplicity of use. However, for the tumour sample, some alterations in the basic protocol were employed. Briefly, the tumour sample, that was frozen at −80°C since surgical extraction, was thawed and immediately fixed in 1% formaldehyde for 25 minutes, followed by the addition of 1× glycine solution for 5 minutes, washed in 1× PBS twice, frozen in liquid nitrogen, and finally pulverized mechanically. The following steps were the same used for breast cancer cell lines.

### Statistical Analysis

Data are expressed as mean values of at least three independent experiments ± s.d. Student's t-tests were used to determine statistically significant differences (*P<0.05).

## Results

### P-cadherin is co-expressed with C/EBPβ and is regulated by this transcription factor in breast cancer cells

**Figure 1:**
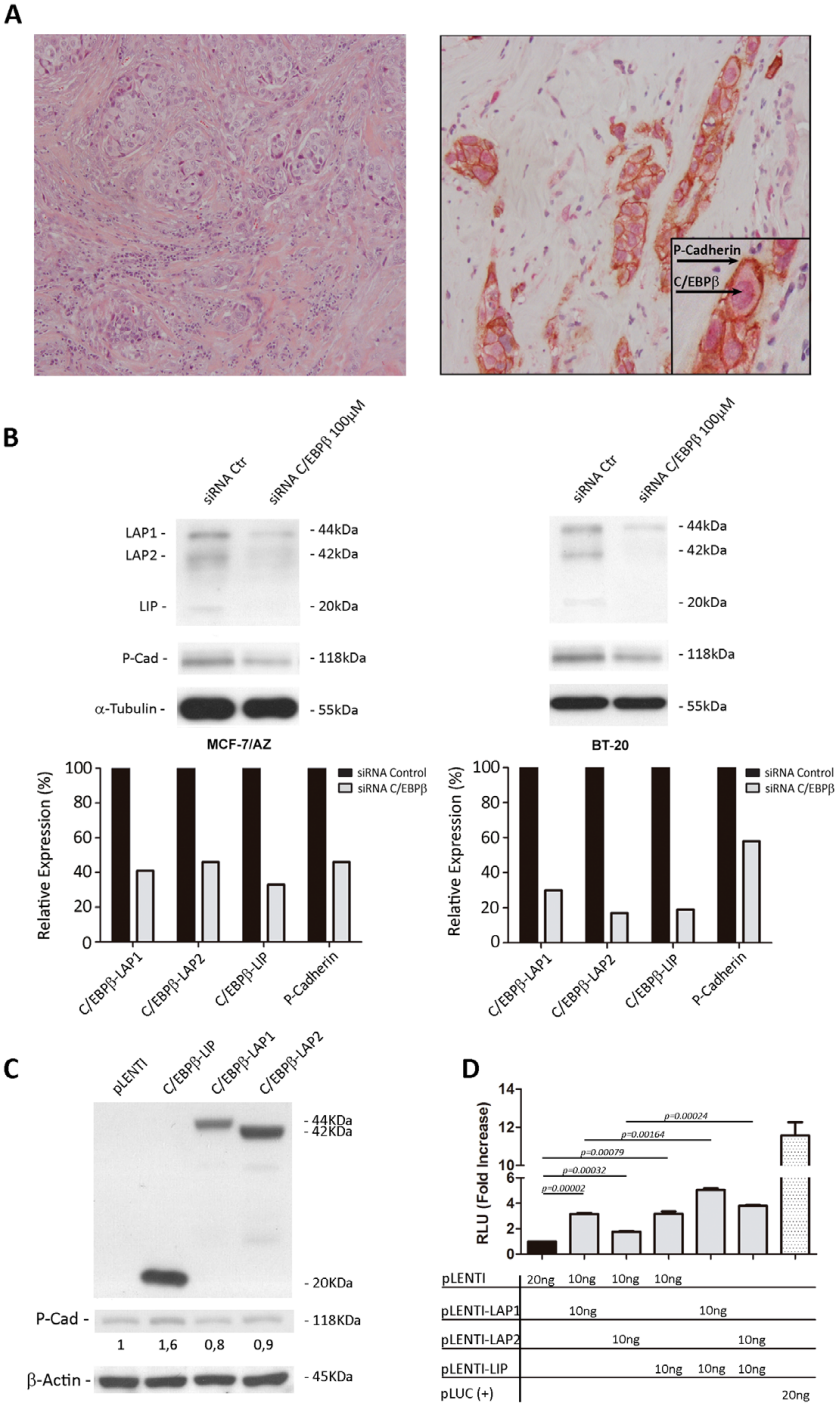
Association and regulatory interplay between C/EBPβ and *CDH3*/P-cadherin expression in breast cancer cells. **A**) Double immunostaining for C/EBPβ and P-cadherin of an invasive breast carcinoma specimen (basal-like carcinoma, histological grade III), where it can be observed C/EBPβ expression in the nuclei and P-cadherin at the cell membrane of tumour cells (magnification ×200 and ×400-inset); a haematoxylin-eosin staining of this same case is shown to ascertain tissue integrity (magnification ×100); **B**) Using C/EBPβ-targeted siRNA, a consequent reduction of P-cadherin protein levels was observed in both MCF-7/AZ and BT-20 breast cancer cell lines; **C**) MCF-7/AZ cells transiently transfected with the different C/EBPβ isoforms (LAP1, LAP2 and LIP) displayed upregulation of P-cadherin protein levels only after induction of the C/EBPβ-LIP isoform; **D**) Luciferase reporter assays performed in cells transfected with the different C/EBPβ isoforms showed that the promoter activation induced by LIP and LAP1 isoforms was significantly greater compared with the activation induced by LAP2. The co-transfection of both LIP and each LAP1 or LAP2 induced the activation of the *CDH3* promoter in an additive manner.

Using a large cohort of invasive breast carcinomas, the expression of C/EBPβ was previously demonstrated to be significantly associated with P-cadherin expression in about 60% of the cases [18]; however, the cellular co-expression of these two proteins was not verified. Thus, based on the hypothesis that C/EBPβ directly activates the *CDH3* gene promoter, a double immunostaining was performed in all invasive breast carcinomas that previously showed strong positivity for both proteins. As represented in Figure 1A, C/EBPβ expression was found in the nuclei of the same cells that were expressing P-cadherin at the cell membrane, pointing for a putative functional relationship between both proteins.

Based on these results, two different breast cancer cell models were used to demonstrate if P-cadherin expression could be affected by C/EBPβ: 1) MCF-7/AZ, which is an ER+/luminal type breast cancer cell line expressing moderate levels of P-cadherin, and 2) BT-20, an ER-negative/basal-like breast cancer cell line, highly positive for P-cadherin [17]. The siRNA mediated-knock-down of C/EBPβ induced a significant downregulation of all C/EBPβ isoforms (LAP1, LAP2 and LIP) in both cell lines. Interestingly, P-cadherin expression was also affected by the reduction of C/EBPβ isoforms, being this effect more pronounced in MCF-7/AZ cells (Figure 1B). According with these results, and in order to decipher which C/EBPβ isoform was more relevant for P-cadherin activation, the expression of LAP1, LAP2 and LIP was induced in both breast cancer cell lines. As shown in Figure 1C, only C/EBPβ-LIP isoform was able to induce P-cadherin expression in more than 1.5-fold increase in MCF-7/AZ cells, while the remaining isoforms did not produce valuable effects on P-cadherin expression. This result was not found for BT-20 cells, probably due to their high basal levels of P-cadherin expression (data not shown).

Interestingly, in a previous study performed by our group, we found that the *CDH3*/P-cadherin promoter activation induced by the LIP isoform was significantly greater compared with the activation induced by LAP1 and LAP2 [18]. However, in the present study, this same experiment has been performed and, although the same significant result was observed at the promoter level for LIP (*p = 0.00079*), the *CDH3* promoter was also strongly and significantly activated by LAP1 (*p = 0.00002*) and less prominently, but also in a significant way, by LAP2 (*p = 0.00032*) (Figure 1D). Nevertheless, since it has been described that LIP can function as a dominant negative inhibitor of both LAP's activity [5], we decided to co-transfect both LIP and each LAP1 or LAP2 , in order to study their combined effect on *CDH3* promoter activity. The results showed that there is a significant increased activation of the promoter with any of the combinations compared with LAP1 or LAP2 alone, demonstrating that there is an additive effect of both isoforms (*p = 0.00164* and *p = 0.00024*, respectively) on *CDH3* promoter activation, when added to LIP.

### C/EBPβ physically interacts with endogenous *CDH3* gene promoter in breast cancer cells

**Figure 2:**
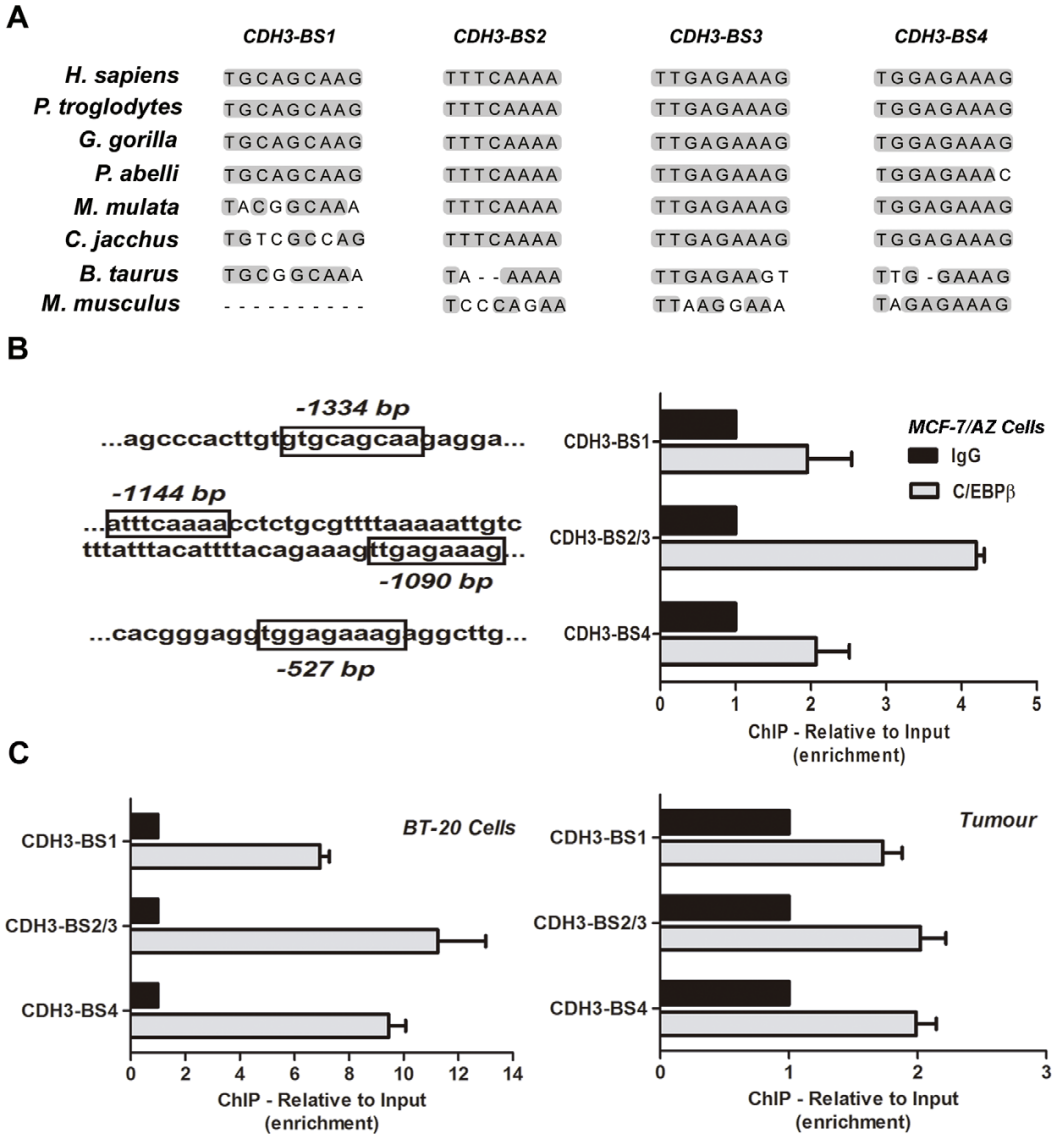
C/EBPβ physical interaction with the *CDH3* gene promoter. **A**) Putative C/EBPβ-binding sites within the *CDH3* gene promoter, where it can be observed their degree of conservation between human and other primates. Grey regions represent total sequence conservation in comparison with human sequence; **B**) Proximal regulatory region of *CDH3* promoter displaying the relative localization of the predicted C/EBPβ binding sites (left panel). The right panel illustrates the enrichment (relative to input) of the *CDH3* promoter DNA-amplified fragments precipitated from DNA-protein complexes obtained by ChIP in MCF-7/AZ breast cancer cells. **C**) ChIP experiment performed in BT-20 breast cancer cells and on a frozen primary breast tumour, highly positive for P-cadherin and C/EBPβ expression, also showed the same enrichment pattern for all the putative binding sites.

Since the three C/EBPβ isoforms were able to transactivate the 1.8 Kb *CDH3* promoter gene construct (Figure 1D), we decided to evaluate in detail the sequence of this putative regulatory region using distinct bioinformatic tools, which can predict for the binding of specific transcription factors. Four concordant C/EBPβ-putative binding sites were identified within the first 1400 nucleotides. Interestingly, we found that there is a high degree of conservation of these predicted C/EBPβ binding sites between humans and other primates within the *CDH3* promoter (Figure 2A), and the left panel of Figure 2B shows their relative localization.

In fact, in order to demonstrate if there was a physical interaction between C/EBPβ proteins and *CDH3* promoter in these specific binding sites, ChIP has been performed in MCF-7/AZ breast cancer cells. Indeed, The results showed that there was an enrichment (relative to *input*) of the *CDH3* DNA-amplified fragments precipitated with the C/EBPβ antibody in all binding sites (Figure 2B, right panel), demonstrating that C/EBPβ transcription factors directly bind to the selected regions within the *CDH3* promoter.

This same experiment has been performed in BT-20 breast cancer cells, as well as in a frozen primary basal-like breast carcinoma, which was selected for being highly positive for P-cadherin and C/EBPβ expression. Interestingly, we could confirm the results, since there was precipitation with the C/EBPβ antibody in all the binding sites studied, in both cells and primary tumour (Figure 2C). Moreover, in BT-20 cells, which overexpress P-cadherin, the binding in all sites was very strong compared with the one found in MCF-7/AZ breast cancer cells.

### C/EBPβ binding sites are important for *CDH3* gene activity and are selectively activated by the different C/EBPβ isoforms

**Figure 3:**
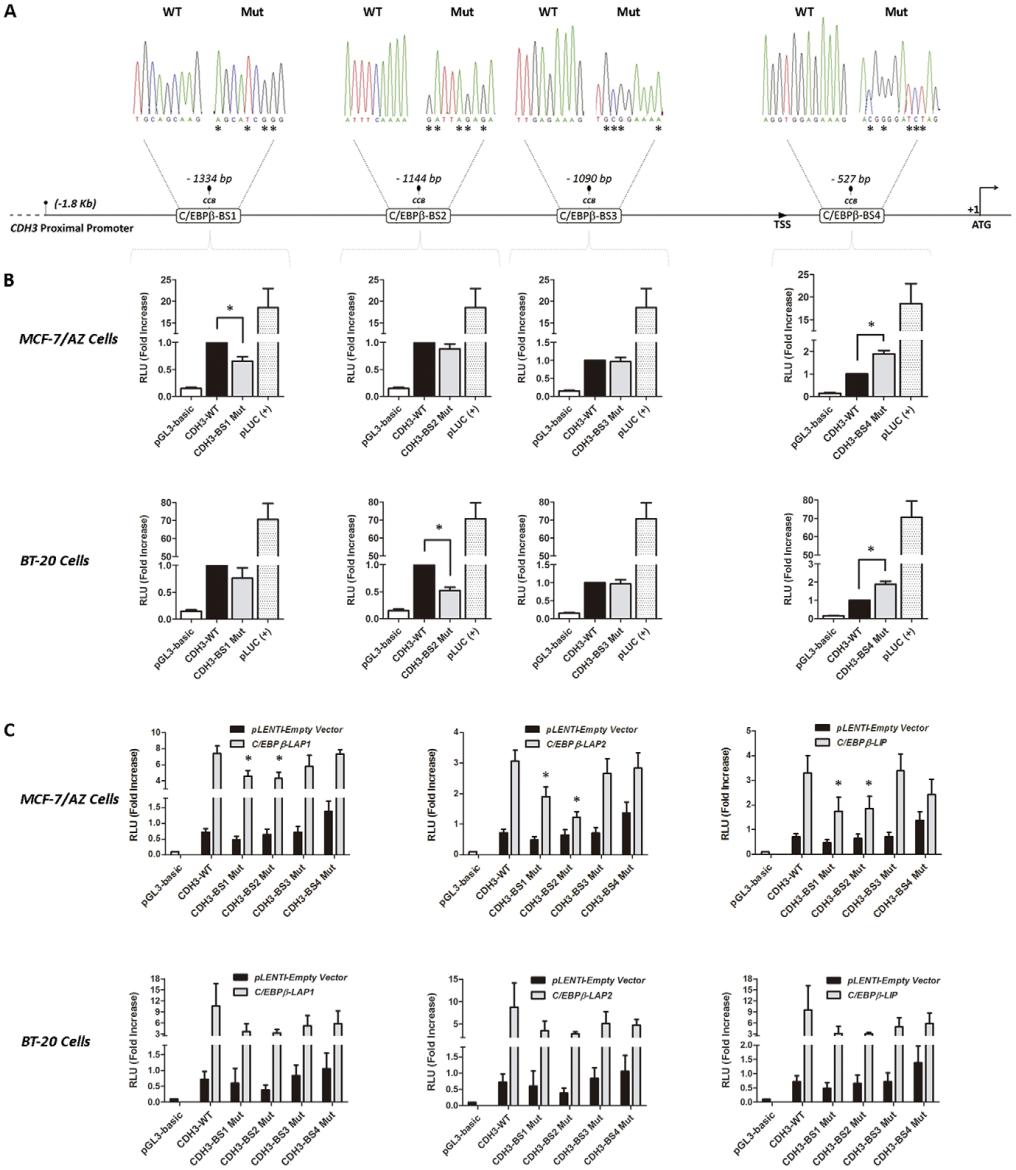
Relevance of C/EBPβ-isoforms and their putative binding sites in the activation of the *CDH3* gene. **A**) Schematic representation of the wild-type and mutated *CDH3* promoter; **B**) *CDH3*-Luciferase Reporter Assays performed with each of the mutations introduced at C/EBPβ binding sites demonstrating that *CDH3*-BS1, BS2 and BS4 are the most important for the activity of *CDH3* promoter in both MCF-7/AZ and BT-20 breast cancer cells; *p-value<0.05; **C**) *CDH3*-Luciferase Reporter Assays upon co-transfection of LAP1, LAP2 and LIP C/EBPβ isoforms, showing the relevance of specific C/EBPβ isoforms across *CDH3* promoter binding sites in both MCF-7/AZ and BT-20 breast cancer cells. *CDH3*-BS1 and BS2, but not BS3 and BS4, are responsive to all C/EBPβ isoforms; *p-value<0.05.

In order to evaluate the importance of the aforementioned binding sites to the *CDH3* gene activation, as well as the specificity of the different C/EBPβ isoforms to the *CDH3* promoter, point mutations were introduced in the specific C/EBPβ binding sequences. Figure 3A illustrates the *CDH3* point mutations and their position within the C/EBPβ binding sites in relation to the wild-type *CDH3* promoter.

Interestingly, when MCF-7/AZ cells were transfected with the *CDH3* promoter containing point mutations at the binding sites 1 and 4 (*CDH3*-BS1 and BS4), there was a statistically significant alteration in *CDH3* promoter activity related to the wild-type promoter sequence (Figure 3B). In contrast, the activity of the *CDH3* promoter was not affected by the mutation introduced at the BS3 site, and only slightly affected by the introduced mutation at the binding site 2 (BS2). These results were mostly confirmed in BT-20 cells, especially for the BS4 mutation, located at the transcription start site region of the *CDH3* promoter, which also significantly induced its activity (Figure 3B). Although not significant, the reduction on *CDH3* promoter activity observed with the BS1 mutant was also found in BT-20 cells, suggesting that this distal C/EBPβ binding site is also important to *CDH3* gene transcriptional activation. In addition, the BS2 mutant significantly reduced *CDH3* promoter activity in BT-20 cells, showing that this is also a crucial site for the activation of P-cadherin transcription in this model. Finally, we could not find any effect of BS3 mutation in *CDH3* promoter activity also in BT-20 cells, proving that this site is not relevant for its regulation.

Since the distinct C/EBPβ isoforms have been documented has having different functions in cancer gene activation and in a cell-specific context, we co-transfected LAP1, LAP2 and LIP together with the different mutants of *CDH3* promoter in both breast cancer cell lines. The results demonstrated that distal *CDH3*-BS1 and BS2 are significantly important for the induced promoter activity mediated by all C/EBPβ isoforms. In contrast, BS3 did not play a significant role in C/EBPβ-mediated *CDH3* promoter activity, since mutations in this specific region were not important to impair the activation of *CDH3* gene mediated by any of the distinct isoforms. Similar results were observed concerning BS4, which did not reveal to be important for *CDH3* promoter activity mediated by LAP1, LAP2 or LIP isoforms. Finally, although not significant, the same trend was observed with BT-20 cells, proving that BS1 and BS2 are most likely the binding sites where all C/EBPβ isoforms bind to induce P-cadherin transcription in breast cancer.

## Discussion

P-cadherin has been receiving a growing interest in the last years, since its overexpression is significantly associated with high histological grade breast tumours and with short-term patient overall survival [11], [23]–[25]. The important association between P-cadherin expression and well-established markers correlated to breast cancer poor prognosis, such as high levels of Ki-67, epidermal growth factor receptor (EGFR), cytokeratin 5 (CK5), vimentin, p53 and HER2, has been also largely documented [11]. Although P-cadherin has been detected as altered in distinct tumour models, its effective role in the carcinogenesis process remains discussible, since it behaves differently depending on the studied cancer cell context [26]. If in some models P-cadherin has been suggested to act as an invasion suppressor, such as in colorectal cancer [27] or in melanoma [28], in several other models, including breast cancer, P-cadherin behaves as an oncogene, inducing increased tumour cell motility and invasiveness when aberrantly overexpressed [12]–[14], [27], [29]–[31].

However, data concerning *CDH3* gene regulation in breast cancer is still very limited. The induction of *CDH3* promoter activity in breast cancer cells was recently described by our group to be putatively linked to the transcription factor C/EBPβ, as well as P-cadherin and C/EBPβ expression have been reported to be highly associated in human breast carcinomas and linked with a worse prognosis of breast cancer patients [18]. In fact, the expression of C/EBPβ shares interesting biologic and functional features with the ones attributed to P-cadherin expression. Similarly to what has been described concerning C/EBPβ biology, P-cadherin is involved in homeostatic processes, such as cell differentiation, development and embryogenesis [32]. We have recently found that P-cadherin enriched cell populations show enhanced mammosphere forming efficiency (MFE), as well as increased expression of CD24, CD44 and CD49f, already described as normal or cancer stem cell markers. These results allowed to link P-cadherin expression to the luminal progenitor phenotype of the normal breast hierarchy and established an indirect effect of P-cadherin in stem cell biology [33]. Interestingly, these findings come along with observations that C/EBPβ regulates stem cell activity and specifies luminal cell fate in the mammary gland, categorizing C/EBPβ as one of the several critical transcription factors that specifies mammary stem cells fate during mammary gland development [34]. In a breast cancer biology setting, another interesting finding is related to the fact that P-cadherin, like C/EBPβ, is not mutated in breast tumours, but its overexpression has been widely described in a subset of aggressive breast cancers [5]. Importantly, at a clinicopathological level, some C/EBPβ isoforms, especially C/EBPβ-LIP, correlates with an ER-negative breast cancer phenotype, highly proliferative and high grade lesions and poor patient outcome [8], [35]. All these characteristics overlap with the ones observed in highly malignant breast tumours overexpressing P-cadherin.

The present work demonstrates for the first time that P-cadherin and C/EBPβ co-localize in the same breast cancer cells, and that there is a physical interaction between this transcription factor and *CDH3* gene promoter. Herein, in addition to the identification of the promoter binding sites that are relevant for the transcriptional modulation of *CDH3* gene activity by C/EBPβ, we still tested the relevance of the different C/EBPβ isoforms along the *CDH3* promoter.

In fact, we show that C/EBPβ-LIP is the only isoform capable to significantly induce P-cadherin protein expression, confirming in a way the results obtained in our previous study, where a significant activation of the promoter was only revealed for LIP, although LAP1 and LAP2 were also able to activate the promoter. However, in this study, we found that *CDH3* gene is also significantly responsive to LAP1 and slightly to LAP2 isoform at the promoter level. These significant results were probably due to improved transfection efficiencies; however, although LAP1 and LAP2 are activating the gene promoter, supporting the classical knowledge described for these isoforms as transcriptional activators, this might not imply that these isoforms induce functional activity through protein synthesis. In fact, it has been largely discussed that the functionally transactivation potential of each C/EBPβ isoform can be highly modulated, since this ability strongly depends not only on dimer composition formed by C/EBPs, but specially on the partner proteins and responsive elements found in target gene promoters [5]. The fact that LIP activates *CDH3* promoter, leading to protein synthesis, reinforces the emerging evidence that LIP acts as a transcriptional activator of gene expression, challenging the long-standing concept that LIP fashionably functions as a dominant-negative isoform [5]. We also observed that LAP2 was the C/EBPβ isoform that activated *CDH3* promoter in a less extent, which is apparently surprising in light that LAP2 isoform is considered to be the most transcriptionally active C/EBPβ isoform [5]. However, it is also known that, in transformed cancer cells, an increase in LIP expression leads to a reduction in LAP2 activity and, therefore, impair its mediated transcription potential [36].

A novel observation also obtained in this study was the existence of interaction between C/EBPβ proteins to the conserved regions of the *CDH3* gene promoter, identified as C/EBPβ responsive elements. The ChIP results, obtained from the DNA region containing both BS2 and BS3 binding sites, revealed a cumulative increased C/EBPβ antibody-precipitated DNA when compared to individual BS1 and BS4, reinforcing the existence of bounding complexes. This was denoted for both MCF-7/AZ and BT-20 breast cancer cell lines and also for the basal-like tumour studied by *in vivo* ChIP.

Concerning the impact of C/EBPβ binding sites to the *CDH3* promoter activity, we found that BS1, BS2 and BS4 were the most relevant ones, while BS3 was not responsible for the modulation of the *CDH3* promoter. A detailed analysis of the *CDH3* promoter using the Ensemble ENCODE Project, revealed two DNAse Hypersensitive (DHS) sites located around BS1 and BS4 specific sequences, confirming an increased regulatory activity on these specific regions.

Interestingly, one of the most curious effects was the one found at BS4, which is located at the transcription start site region of *CDH3* promoter. In contrast with the distal sites, binding impairment at BS4 significantly induced the activity of *CDH3* promoter. In a first approach, we may hypothesize that specific C/EBPβ proteins are regulating negatively the activity of the promoter through that specific binding site and, upon mutation, this repression is released. However, since we did not find a significant effect mediated by LAP1, LAP2 or LIP when BS4 was mutated, we believe that other factors not C/EBPβ-related are responsible for the negative regulation in this binding site, or the mutation introduced in BS4 generated a sequence which allowed the binding of a transcription factor that is able to activate the *CDH3* gene promoter. Additionally, it is also interesting to note that, although the BS2 mutation did not create a significant decrease in *CDH3* promoter activity in MCF-7/AZ cells, this binding site is important to LAP2-mediated activation, indicating that it may not be endogenously active in these breast cancer cells, but probably highly active in BT-20 cells.

In conclusion, this study contributes to clarify the individual role of C/EBPβ proteins in breast cancer-related *CDH3*/P-cadherin gene, as well as to expand the limited characterization of the mechanisms and players that regulate this pro-invasive protein in breast cancer.

## Supporting Information

Table S1 **Conditions of the primary antibodies.**
(PDF)

Table S2 **Primers sequences used in the different assays.**
(PDF)
