# Supplementary material for: CCAAT/Enhancer Binding Protein β (C/EBPβ) Isoforms as Transcriptional Regulators of the Pro-Invasive CDH3/P-Cadherin Gene in Human Breast Cancer Cells
Source: PLoS One. 2013 Feb 6;8(2):e55749. doi: 10.1371/journal.pone.0055749 (PMC3566012; doi:10.1371/journal.pone.0055749)
Supplement: Table S1 — Conditions of the primary antibodies. (PDF) [file pone.0055749.s001.pdf]

Table S1. Conditions of the primary antibodies.

Antigen	Primary Antibody					Incubation time (min)	Antigen Retrieval Buffer
	Clone	Source	Method	Temperature	Dilution		
**P-cadherin**	56	Mouse	IHC	RT	1:50	60	Tris-EDTA (pH=9.0)
			WB	RT	1:500	60	-
**CEBPβ**	H7	Mouse	IHC	4ºC	1:100	O.N.	Tris-EDTA (pH=9.0)
			WB	RT	1:500	60	-
**Actin**	I19	Goat	WB	RT	1:1000	45	-
**Tubulin**	B512	Mouse	WB	RT	1:10 000	45	-
