# Supplementary material for: CCAAT/Enhancer Binding Protein β (C/EBPβ) Isoforms as Transcriptional Regulators of the Pro-Invasive CDH3/P-Cadherin Gene in Human Breast Cancer Cells
Source: PLoS One. 2013 Feb 6;8(2):e55749. doi: 10.1371/journal.pone.0055749 (PMC3566012; doi:10.1371/journal.pone.0055749)
Supplement: Table S2 — Primers sequences used in the different assays. (PDF) [file pone.0055749.s002.pdf]

Table S2. Primers sequences used in the different assays.

**C/EBPβ Cloning**	
**C/EBPβ-LAP1**	F: 5' CAC CAT GCA ACG CCT GGT GGC 3'<br>R: 5' CTA GCA GTG GCC GGA GGA G 3'
**C/EBPβ-LAP2**	F: 5' CAC CAT GGA AGT GGC CAA C3'<br>R: 5' CTA GCA GTG GCC GGA GGA G 3'
**C/EBPβ-LIP**	F: 5' CAC CAT GGC GGC GGG CTT CCC 3'<br>R: 5' CTA GCA GTG GCC GGA GGA G 3'
***CDH3* Promoter Site-Directed Mutagenesis**	
CDH3*-BS1 *Mut	F: 5' CAG AGC CCA CTT GTG AGC ATC GGG AGG AAA GCA CTT C 3'<br>R: 5' GAA GTG CTT TCC TCC CGA TGC TCA CAA GTG GGC TCT G 3'
CDH3*-BS2 *Mut	F: 5' CAG TAT TTC AGA CCA GAT TAG AGA CCT CTG CG TTT TAA AAA TTG TC 3'<br>R: 5' GAC AAT TTT TAA AAC GCA GAG GTC TCT AAT CTG GTC TGA AAT ACT G 3'
CDH3*-BS3 *Mut	F: 5' CAG AAA GTT CGG AGA ATGTTA TTT ATA TGG GGG GTA GGG G 3'<br>R: 5' CCC CTA CCC CCC ATA TAA ATA ACA TTC TCC GAA CTT TCT G 3'
CDH3*-BS4 *Mut	F: 5' CTG GCA CGG GAC GGT GAT CTA GAG GCT TGG GCG G 3'<br>R: 5' CCG CCC AAG CCT CTA GAT CAC CGT CCC GTG CCA G 3'
***CDH3* amplification and Sequencing Screening**	
***CDH3*-BS1**	F : 5' GCT TGT GGG GTA GTG ATT GAG A 3'<br>R : 5' GAC CTG GGA TTC GTT GTT CTT C 3'
***CDH3*-BS2/3**	F : 5' CAG CAA GCA GTT TTT ACC CAG A 3'<br>R: 5' TGC GAG CTA AGA GGG TTG TTA TT 3'
***CDH3*-BS4**	F: 5' GTC CTT CGG GAC CTG CTA GTT T 3'<br>R: 5' GAG GCG CTG TTA TCC CAA AG 3'
